# Hyperscanning: from inter-brain coupling to causality

**DOI:** 10.3389/fnhum.2024.1497034

**Published:** 2024-11-13

**Authors:** Andrey Markus, Simone G. Shamay-Tsoory

**Affiliations:** ^1^School of Psychological Sciences, University of Haifa, Haifa, Israel; ^2^The Integrated Brain and Behavior Research Center, Haifa, Israel

**Keywords:** hyperscanning, structural equation model, causality - causal modeling, neurofeedback, functional near infrared spectroscopy (fNIRS)

## Abstract

In hyperscanning studies, participants perform a joint task while their brain activation is simultaneously recorded. Evidence of inter-brain coupling is examined, in these studies, as a predictor of behavioral change. While the field of hyperscanning has made significant strides in unraveling the associations between inter-brain coupling and changes in social interactions, drawing causal conclusions between brain and behavior remains challenging. This difficulty arises from factors like the inherently different timescales of behavioral responses and measured cerebral activity, as well as the predominant focus of existing methods on associations rather than causality. Specifically, a question remains as to whether inter-brain coupling between specific brain regions leads to changes in behavioral synchrony, or vice-versa. We propose two novel approaches to addressing this question. The first method involves using dyadic neurofeedback, wherein instances of inter-brain coupling are directly reinforced. Such a system could examine if continuous changes of inter-brain coupling are the result of deliberate mutual attempts to synchronize. The second method employs statistical approaches, including Granger causality and Structural Equation Modeling (SEM). Granger causality assesses the predictive influence of one time series on another, enabling the identification of directional neural interactions that drive behavior. SEM allows for detailed modeling of both direct and indirect effects of inter-brain coupling on behavior. We provide an example of data analysis with the SEM approach, discuss the advantages and limitations of each approach and posit that applying these approaches could provide significant insights into how inter-brain coupling supports crucial processes that occur in social interactions.

## Introduction

Neuroscience is largely dedicated to studying the brain’s structure and function, with a primary focus on understanding how neural processes influence behavior, cognition, and overall mental health. To this aim, traditional neuroimaging studies have relied on assessing activation estimates within specific regions of interest (ROIs) and examining their relationship with behavior (e.g., Molenberghs et al., 2016). As cognitive processes are often the result of complex interactions between multiple regions, not the activity of single areas in isolation (Bullmore and Sporns, 2009; Damoiseaux et al., 2006; Smith et al., 2009), research has shifted toward utilizing network-level brain variables to explore brain-behavior relationships, reflecting the interdependent nature of neural processes (Bassett and Sporns, 2017).

A recent approach in the field of social neuroscience has extended the network approach to social interactions, suggesting that brain activity is not only coupled within an individual brain but is also coupled between brains during social communication. According to the hyper-brain cell assembly hypothesis, neural cell assemblies may form not only within individual brains but also across brains, operating under principles similar to Hebbian learning within a single brain (Müller, 2022). Within this framework, inter-brain coupling refers to the correlation of time series of brain signals originating from regions of two or more interacting brains (Dikker et al., 2021). The technique of measuring inter-brain coupling is called hyperscanning, an approach that allows simultaneous scanning of multiple brains (Montague et al., 2002). Hyperscanning employs neuroimaging techniques such as Electroencephalography (EEG), functional Magnetic Resonance Imaging (fMRI), and functional Near-Infrared Spectroscopy (fNIRS) and it is increasingly used to examine joint brain activity in individuals within social interactions in various social paradigms. For example, in typical hyperscanning fNIRS studies participants are assigned into dyads or groups and are asked to perform a joint task, while inter-brain coupling in fNIRS signals are taken from all participants. Given the relatively mobile, robust and unintrusive nature of the available portable fNIRS systems, a wide range of interactive tasks can be used in ecologically valid environments, including motor, emotional and cognitive tasks, as well as creativity and problem-solving tasks. Inter-brain coupling values from the obtained fNIRS data are calculated post-recording, and often compared against other, concurrently obtained, task-related data, such as synchrony in speech, eye-movements or motor activity.

The findings of inter-brain coupling during social interactions have greatly advanced neuroscience by demonstrating that multiple brains of interacting individuals can be viewed as components of an extended network (Shamay-Tsoory, 2022). With this approach studies have shown for example that inter-brain coupling in the inferior frontal gyrus (IFG) is increased during face-to-face interaction compared to no-interaction (Jiang et al., 2012), during synchronized movement (Gamliel et al., 2021) and during song learning (Pan et al., 2018). Other brain regions including the dorsolateral prefrontal cortex and the temporoparietal regions were shown to be highly coupled during tasks of group creativity (Mayseless et al., 2019; Pick et al., 2024) and group collaboration (Xie et al., 2020).

However, the initial enthusiasm from hyperscanning was tempered by concerns that inter-brain coupling might merely be an epiphenomenon of performing the same activity simultaneously (Hamilton, 2021). To address this issue, new statistical approaches have been developed, including demonstrating that inter-brain coupling is stronger in real interacting pairs compared to pseudo-pairs [non-interacting pairs performing the same task (Marton-Alper et al., 2023)] or showing that inter-brain coupling is not entirely explained by motor synchrony (Pérez et al., 2017). In a study with dyads of rodents, it was demonstrated that inter-brain coupling emerges from two neuronal populations that separately encode one’s own behaviors and those of the interaction partner, providing evidence that inter-brain coupling arises from ongoing exchange of social signals (Kingsbury et al., 2019). Furthermore, inter-brain coupling has been shown to yield higher predictive power for learning outcomes during social learning compared to single-brain measures (Davidesco et al., 2023), emphasizing the importance of incorporating these measures into models of social behavior.

Despite these exciting developments, a critical question remains regarding the causal relationship between inter-brain coupling and behavioral change. While hyperscanning studies to date have examined the association between inter-brain coupling and behavior, it is not yet clear whether inter-brain coupling triggers behavioral changes or if dyadic behavior creates inter-brain coupling (Hamilton, 2021). It could be the case that during coordinated activities (e.g., joint actions), individuals are exposed to the same sensory stimuli, such as visual or auditory cues. These shared inputs can result in similar neural responses in the brains of the individuals involved, leading to coupled neural activity. Furthermore, during coordinated behavior, individuals often predict each other’s actions and adjust their own actions accordingly. This anticipatory mechanism involves neural processes that align the timing of neural activity between brains and could lead to inter-brain coupling. Yet, another possibility is that inter-brain coupling modulates behavior, with fluctuations in coupling levels driving the dynamics of communication during social interactions. Given that social interactions inherently involve a continuous feedback loop of reciprocal exchanges, this bidirectional interaction may underlie the causal relationship between inter-brain coupling and behavioral outcomes. Here, we emphasize the importance of examining the causal pathways between brain activity and behavior, as understanding this relationship is crucial for elucidating the neural mechanisms underpinning inter-brain coupling. Such an investigation could reveal how neural coupling promotes effective communication and facilitates social coordination. We propose here two approaches for testing causality: The first approach relates to manipulating the brain with neurofeedback and involves providing real-time feedback to participants based on their inter-brain coupling. The second approach offers statistical methods for assessing causality. We discuss these two options including the advantages and limitations of each approach and their feasibility in addressing mechanistic explanation of social behavior.

## Dyadic neurofeedback

While emerging studies with brain stimulation targeting the IFG show a causal relationship between simultaneous dyadic IFG stimulation and increased coupling (Novembre et al., 2017), it is unclear whether inter-brain coupling could be trained and to what extent training is translated into behavioral change.

Neurofeedback is a technique that provides real-time information about the current level of brain activity, to which we otherwise do not have conscious access. Such information can be used to learn volitional regulation of brain activity. The feedback is visual or auditory (e.g., an animated fish swimming in the sea), in which changes in certain parameters (e.g., the fish’s movement) reflect changes in certain features of the measured brain activity. Neurofeedback leverages the brain’s ability to reorganize itself following operant conditioning (Paret et al., 2019) and Hebbian-like plasticity (Coscia et al., 2019) by selectively reinforcing specific neuronal changes.

**FIGURE 1:**
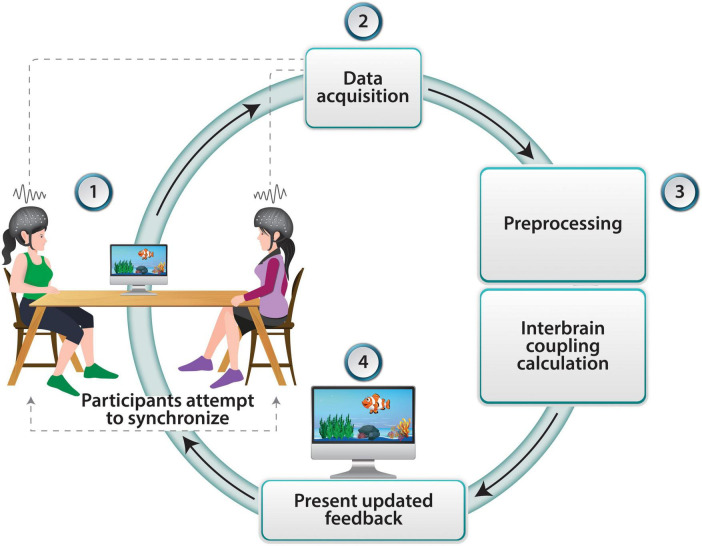
Neurofeedback loop in dyadic interaction using fNIRS. Two participants observe a swimming fish that and its velocity represents their levels of inter-brain coupling. fNIRS data is collected from both participants in real-time as they perform the task. The recorded fNIRS data undergo preprocessing to remove artifacts and noise. Interbrain coupling is then calculated and the real-time inter-brain coupling information is presented back to the participants through updated movement of the fish, thus completing the neurofeedback loop.

In the same manner that neurofeedback can regulate the activity of specific brain regions, training with a dyadic neurofeedback platform may allow participants to control their inter-brain coupling by providing feedback on this coupling. Such a setup could involve reinforcement via a visual signal following increased inter-brain coupling between selected regions (see Figure 1). Initial attempts have demonstrated the feasibility of connecting two participants in a single feedback loop. For example, using EEG, Chen et al. (2021) showed an association between social closeness and inter-brain coupling in a dyadic neurofeedback protocol. While no study to date has demonstrated long-term behavioral changes following dyadic neurofeedback training, the growing efforts to develop such protocols demonstrate significant potential.

A dyadic neurofeedback setup may include simultaneous synchronized fNIRS data collection from two participants. The data obtained from each participant are pre-processed in real-time by application of frequency filtering, motion artifact removal, and common component removal using Short Separation channels (Gagnon et al., 2012) or other statistical methods (e.g., Zhang et al., 2016). The filtered data is then separated into temporal windows of 30 s or more, and inter-brain coupling values are extracted from each window using the Wavelet Transform Coherence (WTC) technique (Grinsted et al., 2004) that enables transforming a time series into a function of time and frequency. This process repeats in intervals of approximately 1 s, with each output value representing the brain synchrony within the preceding window, with a large degree of overlap. These values may be used to adjust the motion speed of an animated fish on a screen, which the participants in the study can observe. Participants are randomly assigned to dyads and instructed to observe a computer screen displaying a virtual task involving a swimming fish (Figure 1). Their goal is to increase the speed of the fish’s movement. The speed of the fish is contingent upon the participants’ ability to enhance their inter-brain coupling, providing real-time reinforcement of their increasing interbrain coupling. This feedback loop is designed to promote enhanced synchronization between the participants’ brain activity, thereby facilitating functional inter-brain connectivity. If indeed it will be possible to measure noticeable behavioral change following training in dyadic neurofeedback, it will provide initial evidence that indeed inter-brain coupling supports behavioral change. The changes could be observed at the individual level, for example increased empathic capacities of a participant following dyadic training, or changes at the level of the dyad, such as improved motor synchronization or enhanced cooperation.

The application of dyadic neurofeedback offers several distinct advantages. Unlike neurostimulation, neurofeedback is a non-invasive technique, making it a safer and more accessible option for modulating brain activity. In addition to its potential for testing causal inferences in neural circuits, successful implementation of dyadic neurofeedback could lead to a wide range of clinical applications aimed at enhancing social behavior in various populations.

Despite the potential of dyadic neurofeedback for establishing causal links between inter-brain coupling and behavioral changes, significant challenges remain in developing appropriate control conditions to effectively rule out placebo effects and expectation biases. Additionally, there are unresolved questions regarding the specific brain regions that are most relevant for these dyadic neurofeedback protocols. While a recent meta-analysis found the largest effects size for inter-brain coupling in the frontal cortex and temporoparietal junction (Czeszumski et al., 2022), it is possible that other brain regions may also have potential for training inter-brain coupling. In addition, the process of learning to control brain activity within dyadic neurofeedback is inherently slow and complex. Finally, variability in individual learning abilities within the dyad can significantly influence the overall effectiveness of achieving and maintaining enhancements in inter-brain coupling. Nonetheless, demonstrating that modifications in inter-brain coupling can lead to measurable behavioral changes would provide compelling evidence of causality, reinforcing the potential impact of such interventions on social and cognitive processes.

## Statistical approach for testing causality

Various statistical tools have been employed to address the challenge of causality in neuroimaging studies, including Granger causality and structural equation modeling (SEM). These methodologies can be applied to explore the causal dynamics of inter-brain coupling and its impact on social interactions, thereby providing insights into the neural mechanisms underlying social behavior.

The Granger causality approach relies on the principle of comparing the predictive power of a past value of a variable Y on its current value (autoregression) to the predictive power of autoregression and the values of a second variable X. If the latter is significantly higher than the former, X can be said to be G-causing Y (Seth et al., 2015). Importantly, Granger causality tests can be performed in the opposite direction – i.e., whether Y is G-causing X. Significance in both cases is not mutually exclusive. If changes in the neural activity of one brain can be shown to G-cause changes in another brain’s activity, and this neural interaction correlates with specific social behaviors, it becomes possible to predict how changes in inter-brain coupling may influence or correspond to future behaviors in social interactions. G-causality has been extensively utilized in neuroimaging studies, particularly within the framework of ordinary linear autoregressive (AR) models of stochastic processes (Goebel et al., 2003; Roebroeck et al., 2005), and it may therefore become valuable in understanding and forecasting the dynamics of social behavior based on inter-brain coupling. One possibility is employing Granger causality to investigate the directionality between inter-brain and intra-brain coupling. Indeed, Müller and Lindenberger (2024) showed that the dynamics of both inter-brain and intra-brain connections are critical for understanding interpersonally coordinated actions. If it is found that inter-brain coupling influences intra-brain connectivity, this would suggest that changes in inter-brain dynamics may drive alterations in individual neural processes, ultimately leading to behavioral modifications.

To establish causal link between inter-brain coupling and behavior, Koul et al. (2023) used synchronized EEG recordings from dyads engaged in a nonverbal task. The authors applied G-causality explore the directional relations between inter-brain coupling and behavioral measures (e.g., synchrony in facial expressions). To this end, participants were placed at either a short (1 m) or long (3 m) distances from each-other under conditions where they could or could not see each-other, under no specific instructions to interact. During each block, EEG, movements and eye-tracking recordings were obtained. Inter-brain coupling emerged spontaneously when participants were looking at each-other regardless of distance. The authors first analyzed the power spectra across several EEG frequency bands. They then used computational model, which relied on contemporaneous power increase in these frequency bands within dyads, to identify instances of inter-brain coupling. Behavioral recordings were analyzed to produce a matching timeline, representing synchronous behavior. These timelines were then used to construct a Bayesian model, which was then fitted to a vector autoregressive (VAR) model, which, in turn, was used to calculate multivariate G-causality values. G-causality was found significant in both directions, although the predictive effects of behavioral synchrony on inter-brain coupling were reported to be stronger than the opposite (Koul et al., 2023). The latter finding raises questions about the specific mechanism of this reciprocity. It may be hypothesized, for example, that inter-brain coupling between specific ROIs may causally affect behavioral coupling, and vice-versa for other ROI pairs. Exploration of this topic is somewhat hampered by the difficulty in source localization inherent in EEG recordings (e.g., Bradley et al., 2016; Jatoi et al., 2014). In contrast, techniques relying on cerebral haemodynamic response, such as fMRI and fNIRS, which are relatively accurate in their spatial resolution, may lack the required temporal resolution for G-causality analyses. For example, in a typical study using fNIRS for measuring brain activity, the haemodynamic response may be trailing the underlying neural activity by several seconds (Cinciute, 2019), thus leading to the possibility of the resulting behavior taking place before the haemodynamic response can be recorded. One possibility is to use simultaneous recordings of fNIRS and EEG data which may allow identifying causal relationships between specific ROI pair coactivation and behavioral synchrony in more detail.

Another potential approach toward establishing and testing network-based models of brain and behavior may be based on structural equation modeling (SEM). In SEM parameters are represented by connection strengths or path coefficients between variables, analogous to effective connectivity in a neural network model. Each path within the model is directional, reflecting hypothesized causal influences between variables. The parameters in SEM are estimated by minimizing the discrepancy between the observed covariance matrix and the covariance matrix predicted by the proposed structural model. This estimation process enables SEM to solve the entire path model simultaneously, providing insights into the causal directionality among multiple ROIs. In a study modeling both brain and behavior, Bolt et al. (2018) conducted a study on data from a large pool of participants, who performed tasks from three cognitive domains (working memory, relational processing, and arithmetic processing), with each task having a respective control condition. fMRI recordings were taken during the tasks’ completion. The authors then used SEM to construct a network model, based on the collected data, which included several ROIs in the brain as well as behavioral responses. A regression equation was constructed to represent the activity of each ROI as a function consisting of a network activation component, an ROI activation component unrelated to the network, and a residual component. The behavioral outcome was then estimated as a regression function including the activity within the network, the network-independent activity in each ROI, and a random component. The resulting model was then pruned to remove ROIs with low loadings. Using this method, only individual ROIs were shown to affect behavior independently of the network as a whole. This approach is highly promising for application in inter-brain coupling. In particular, it allows for testing of specific hypothetical models, such as models that include inter-brain coupling between specific regions and behavior.

**FIGURE 2:**
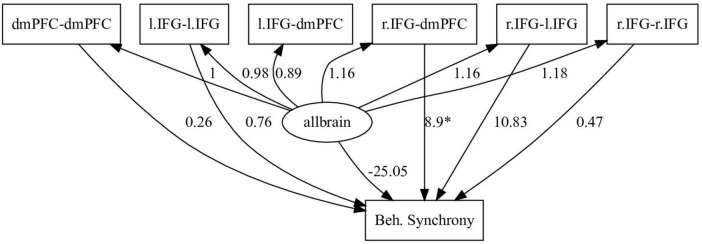
Example SEM model. Predicting behavioral synchrony by the measured activity of several ROI couplings and the computed combined activity of the interbrain network as a whole (allbrain). Here, only couplings including r.IFG and dmPFC were used to predict behavior, according to previous findings. The loading values are indicated by the numbers, while significance is indicated by the asterisk. Only the r.IFG-dmPFC coupling was shown to significantly positively predict behavioral synchrony.

We tested this approach using an fNIRS dataset published by Marton-Alper et al. (2023), where we previously found that interbrain coupling in the right inferior frontal gyrus (r.IFG) and dorsomedial prefrontal cortex (dmPFC) positively predicted movement synchronization during a 3D movement task. In this study, dyad members were handed a RAZER 3D motion sensor and instructed to move their arms in synchrony. The 3D position data of the participants’ arms were recorded, and fNIRS inter-brain coupling data were obtained from each participant’s dmPFC and IFG areas, bilaterally. The levels of motion synchrony were calculated as a timeseries throughout each task block by means of Cosine Velocity Vector (CVV) calculation (Reiss et al., 2019), which allows or detection of lagged and unlagged synchronized 3D motion. fNIRS data were preprocessed by frequency filtering, motion artifact removal, PCA-based spatial filtering (Zhang et al., 2016), and converting to relative oxygenated and deoxygenated hemoglobin concentrations. Inter-brain coupling was calculated on the oxygenated hemoglobin values using the WTC toolbox for Matlab (Grinsted et al., 2004) between all ROI combinations. This method of detecting inter-brain coupling is based on a two-stage process, wherein the hemoglobin concentrations are first subjected to wavelet transformation (WT) using a seed Morlet wavelet, which serves the functional purpose of detecting single-brain activity across time and wavelength. We used the wavelengths of 6–66 s. In the second stage, coherence between the two WT series is calculated, to derive WTC values between the two brains. Whereas in the Marton-Alper et al. (2023) study we used LME-type analyses, here we constructed an SEM model using the same data, in which we sought to examine the effects of inter-brain coupling between all measured pairs of ROIs on motion synchrony. The simple assumption behind this model, provided as a feasibility example, was that coupling between some ROIs are likely to be correlated to behavioral synchrony, while inter-brain coupling between others might not. For our purposes, we considered two sets of ROI pairs – each set related to one hemisphere. In our model, overall interbrain connectivity network was represented by a latent variable (allbrain), representing joint effect of all ROI pairs’ activation on behavior, and consisting of the coupling values of all ROIs (l.IFG-l.IFG, l.IFG-dmPFC, l.IFG-r.IFG, r.IFG-dmPFC, r.IFG-r.IFG, dmPFC-dmPFC), and the level of behavioral synchrony (movement synchronization) was predicted by the computed activity of the allbrain variable, and by each of the ROI couplings including r.IFG and dmPFC. As shown in Figure 2, the model indicated that only the r.IFG-dmPFC coupling significantly (*p* < 0.05) predicted behavioral synchrony. A similar model using l.IFG and dmPFC couplings to predict behavioral synchrony levels was tested and yielded no significant predictions. These findings not only corroborate the original findings of Marton-Alper et al. (2023) but also offer a model that includes directionality interdependencies between multiple observed and latent variables.

While we use this as a simple example of using SEMs to describe the relationship between inter-brain and behavioral synchrony, it is possible that data of neural activity as well as social behaviors collected from multiple individuals could be tested by means of multilevel structural equation modeling (mSEM), as proposed by Rabe-Hesketh et al. (2004). In this type of model, ROIs from each individual in a group would be treated as clusters on the lower level of the model, whereas the upper level of the model would represent the group as a whole. Behavioral outcomes can then be factored into such a model in two ways: individual behavior can be factored in as related to the lower-level clusters, respectively; group behavioral measures, such as motor synchrony levels, can be related to both levels of the model.

When comparing the two approaches, the advantage of Granger causality (G-causality) is its ability to provide direct statistical evidence of causality, including the direction of the causal relationship, between continuous time series data. Yet, on its own it provides a fairly narrow view individual connections within the wider network of ROIs involved in inter-brain coupling and the concurrent behavior. In contrast, SEM-based models provide an excellent potential tool for testing the structure of this network as a whole, albeit somewhat limited in inferring causality, in the sense that the direction of causality needs to be hypothesized a-priori in the model being tested. The latter is especially relevant for social interactions which occur in relatively naturalistic settings, as opposed to highly structured trial-based fMRI tasks. We propose that the way forward may be in combining the two approaches.

## Conclusion

Overall, the latest literature on hyperscanning is converging toward the networked approach and much work is being done to devise statistical and experimental methods to validate an overall network model of social interaction. An important aspect of this kind of modeling is the question of causality between groups of ROIs constituting the inter-brain network, and between these groups and the concurrent behavior. Although dyadic neurofeedback studies provide preliminary evidence of causality in neural coupling and its effects, more robust methods are required to thoroughly examine the causal link between inter-brain coupling and behavioral outcomes. Approaches such as Granger causality and SEM offer powerful tools to test the directional influence of inter-brain coupling on behavior. Granger causality is particularly useful for identifying causality of temporal relationship between inter-brain coupling and subsequent behavioral changes. On the other hand, SEM enables researchers to model complex relationships between multiple observed and latent variables, allowing for the simultaneous testing of neural and behavioral dynamics. By applying these techniques, future research could move beyond correlational observations and establish more definitive causal mechanisms linking inter-brain coupling with social and cognitive behavior.

While inferring causal relationships between intra- and inter-brain coupling levels can be achieved using well-established statistical techniques, such as G-causality and SEM, doing the same at the brain-behavior level is more challenging. We propose that application of mSEM modeling together with measures of causality estimation may be highly beneficial for understanding the brain and behavioral dynamics of synchronous behavior. Working from a vantage point of a specific theoretical model of interpersonal interaction, it should be possible to construct and validate its structure and components by examining a statistical mSEM model based on it. Behavioral components may be integrated into this model on multiple levels by means of attributing specific behavioral changes to ROIs that are established as causing behavior directly.

## Data Availability

Publicly available datasets were analyzed in this study. This data can be found here: https://doi.org/10.17632/xd9zr7hh3z.1.
